# The Role of Cytokine Gene Polymorphisms in Rehabilitation Outcome After Traumatic Brain Injury

**DOI:** 10.3390/cells14141056

**Published:** 2025-07-10

**Authors:** Franca Rosa Guerini, Cristina Agliardi, Milena Zanzottera, Antonio Caronni, Laura Antolini, Chiara Camilla Derchi, Tiziana Atzori, Elisabetta Bolognesi, Jorge Navarro, Mario Clerici, Angela Comanducci

**Affiliations:** 1 Laboratory of Molecular Medicine and Biotechnology, IRCCS Fondazione Don Carlo Gnocchi ONLUS, 20148 Milan, Italy; fguerini@dongnocchi.it (F.R.G.); mzanzottera@dongnocchi.it (M.Z.); lantolini@dongnocchi.it (L.A.); cderchi@dongnocchi.it (C.C.D.); tatzori@dongnocchi.it (T.A.); ebolognesi@dongnocchi.it (E.B.); jnavarro@dongnocchi.it (J.N.); mario.clerici@unimi.it (M.C.);; 2 Department of Neurorehabilitation Sciences, IRCCS Istituto Auxologico Italiano, 20149 Milan, Italy; acomanducci@dongnocchi.it; 3 Department of Biomedical Sciences for Health, University of Milan, 20122 Milan, Italy; 4 Department of Medicine and Surgery, University of Milano Bicocca, 20900 Monza, Italy; 5 Department of Pathophysiology and Transplantation, University of Milan, 20122 Milan, Italy

**Keywords:** cytokine genes, cytokine genes polymorphisms, traumatic brain injury, rehabilitation, head trauma, post-traumatic confusional state

## Abstract

Traumatic brain injury (TBI) affects millions of people worldwide and often results in long-term disabilities. Clinical outcomes vary widely even among patients with similar injury severity, partly due to systemic neuroinflammatory responses mediated by pro- and anti-inflammatory cytokines. Genetic polymorphisms in cytokine-coding genes may influence cytokine expression, thereby affecting rehabilitation and prognosis. We analyzed genetic polymorphisms in the *TNF-α*, *IL-6*, *IL-6* receptor, *IL-1β*, and *IL-10* genes in 28 subacute TBI patients undergoing rehabilitation. Clinical outcomes were assessed using the Glasgow Outcome Scale Extended (GOSE) and domain-specific scales for cognitive, motor, and functional recovery. Results were correlated with genetic profiles to identify potential predictive biomarkers. The *IL-6-174* (GG) and *IL-6R* 1073 (AA) genotypes correlated with worse GOSE scores (*p* = 0.02 and *p* = 0.01, respectively). Co-segregation of *IL-6-174* - *IL-6R 1073* G-A alleles was linked to poorer outcomes (*p* = 0.01). Patients with the *TNF-α-308* (GA) genotype showed less improvement in Barthel and Mobility scores (*p* = 0.001 and *p* = 0.01, respectively) and had a higher incidence of post-traumatic confusional state after rehabilitation (*p* = 0.03). Overall, the *TNF-α-308*(GA), *IL-6 -174*(GG), and *IL-6R 1073*(AA) genotypes negatively impact rehabilitation outcomes, likely due to their role in enhancing neuroinflammation. Larger studies are needed to develop personalized therapies tailored to genetic profiles, aiming to improve rehabilitation outcomes for TBI patients.

## 1. Introduction

Neurotrauma, or traumatic brain injury (TBI), is most commonly caused by motor vehicle accidents, falls, and violence. Millions of individuals, especially young adults and older individuals, are affected every year by TBI. Males are at higher risk, and falls, traffic accidents, and sports injuries are the most frequent causes of TBI, which often results in long-term physical, cognitive, and emotional impairments that significantly impact the quality of life and long-term independence [1,2,3]. The clinical management of TBI involves a range of treatments, including procedures to remove blood clots or relieve intracranial pressure, medications to control seizures or reduce inflammation, and intensive rehabilitation aimed at restoring function. This approach also aims to minimize secondary brain injury, which can occur due to factors such as cerebral edema, hypoxia, and ischemia, and supports the recovery of both physical and cognitive functions [4]. Emerging treatments, such as neuromodulation techniques and pharmacological strategies targeting neuroinflammation and excitotoxicity, are also being investigated to enhance neuroplasticity and improve long-term outcomes in this clinical population [5]. Several factors influence TBI clinical outcomes, including the severity of the injury, comorbid conditions, age, and sex [6]. Despite these efforts, patient outcomes following TBI remain highly variable—even among individuals with similar injury severity and treatment protocols—suggesting the influence of additional biological factors.

Emerging evidence highlights the pivotal role of neuroinflammation in shaping both acute damage and long-term recovery after TBI [7] both by promoting neuronal death, edema, and fever and by influencing long-term repair processes [8]. This inflammatory response, primarily driven by cytokines and variations in the DNA sequences within the genes encoding these cytokines, can alter their production [9]. Pro-inflammatory cytokines, like tumor necrosis factor-alpha (TNF-α), interleukin-1 β (IL-1β), and interleukin-6 (IL-6), are upregulated following brain injury and likely contribute to secondary tissue damage [10,11,12]. On the other hand, anti-inflammatory cytokines, like interleukin-10 (IL-10), have a protective effect, helping to reduce inflammation and promote tissue repair [13].

Functional genetic variations in the promoter regions of genes play a major role in interindividual variability in cytokine production [14,15]. In response to TBI, there is a complex upregulation of cytokine gene expression. TNF-α-308G/A polymorphism was shown to be associated with outcomes at 6 months post-TBI; Nevertheless, the interactions among different cytokines and the balance between pro- and anti-inflammatory mechanisms are key factors in influencing the extent of secondary injury and prospects for recovery [7].

While it is well established that neuroinflammatory processes affect outcomes after TBI, there is limited understanding of how specific genetic polymorphisms in cytokine-related genes modulate recovery during the subacute phase, particularly in the context of intensive rehabilitation.

To address this gap, the study aimed to explore the relationship between genetic polymorphisms in cytokine-related genes and functional recovery outcomes in subacute TBI patients undergoing rehabilitation, in order to identify predictive genetic biomarkers for rehabilitation responsiveness and long-term prognosis. To this end, we analyzed a panel of SNPs in the *TNF-α*, *IL-6*, *IL-6* receptor, *IL-1β*, and *IL-10* genes- cytokines suggested to be involved in the pathophysiology of TBI [16,17]- in patients diagnosed with subacute TBI who were enrolled in an intensive rehabilitation program. 

## 2. Materials and Methods

### 2.1. Clinical Assessment

A cohort of 28 subjects (5 females; mean age: 45.7 ± 17.8 years) with a diagnosis of TBI (mean time since injury: 35.56 ± 18.12 days) in the subacute phase (i.e., less than 3 months) was consecutively recruited at the inpatient Acquired Brain Injury Intensive Rehabilitation Unit (IRU) of IRCCS Fondazione Don Carlo Gnocchi in Milan, Italy. Informed consent was obtained from all the individuals included in this study. All procedures were conducted according to the Declaration of Helsinki guidelines and this study was approved by the institutional ethical committee of the IRCCS Fondazione Don Carlo Gnocchi ONLUS, Milan (Protocol number #02_20/05/2021, project acronym: SABINE).

To ensure clinical homogeneity, the inclusion criteria were as follows: (1) subacute TBI (within three months post-injury); (2) moderate to severe injury severity; and (3) the presence of diffused axonal injury confirmed by neuroimaging.

The exclusion criteria included the following: (1) age < 18 or >75 years; (2) a previous neurological or psychiatric disorder (particularly neurodegenerative or acquired conditions affecting cognitive domains, e.g., dementia); (3) medical instability; and (4) the presence of a disorder of consciousness at admission.

All subjects underwent a structured intensive rehabilitation program over a period of two months.

Patients were assessed both at admission (T0) and at discharge (T1) using a set of standardized clinical scales covering different domains of cognitive, motor, and functional recovery.

Cognitive functions were examined using the Frontal Assessment Battery (FAB) [18], a tool designed to evaluate executive functions such as conceptualization, mental flexibility, motor programming, sensitivity to interference, inhibitory control, and environmental autonomy. Behavioral aspects were examined using, the Agitated Behavior Scale (ABS) [19] which quantifies the presence and severity of agitation, a frequent behavioral disturbance observed in patients with subacute TBI.

Motor and functional recovery were assessed through the Barthel Index (BI) [20], which measures the ability to perform activities of daily living, and the Performance-Oriented Mobility Assessment (POMA) [21], a tool specifically designed to evaluate balance and gait, providing insights into fall risk and overall motor recovery.

Global outcome and disability were evaluated through the Glasgow Outcome Scale Extended (GOSE) [22], which measures overall disability levels, and the Disability Rating Scale (DRS) [23], which assesses cognitive and functional impairment, including consciousness levels, self-care ability, dependence, and employability.

### 2.2. Rehabilitation Protocol

During the post-acute rehabilitation phase, patients with TBI frequently experience a condition known as post-traumatic confusional state (PTCS) characterized by disorientation, cognitive fluctuations, and impaired memory. The presence of this condition was diagnosed using the Galveston Orientation and Amnesia Test (GOAT) [24].

The intensive rehabilitation program included an individualized combination of cognitive therapy, physical therapy, occupational therapy, and speech therapy, with the goal of optimizing functional recovery. In accordance with the International Group of Cognitive Researchers and Clinicians (INCOG) guidelines for cognitive rehabilitation after TBI, neuropsychological therapy was initiated only after the resolution of PTCS. The resolution of PTCS, assessed via the GOAT, corresponded to a specific level of cognitive functioning of at least Level VI (Confused-Appropriate) on the LCF (levels of cognitive functions) [25]. 

### 2.3. Additional Clinical Considerations

To address potential confounding variables that could influence both genetic patterns and rehabilitation outcomes, two additional clinical variables were systematically assessed. Given that systemic infections, including sepsis, can exacerbate neuroinflammation and contribute to neuronal damage and prolonged recovery periods, the occurrence of fever lasting more than one day and requiring antibiotic treatment was documented as a potential confounder. Additionally, the use of nonsteroidal anti-inflammatory drugs (NSAIDs) and corticosteroids, known to modulate inflammatory pathways post-TBI and potentially impact neuroplasticity and rehabilitation, was systematically evaluated in each patient to evaluate their potential effect on recovery trajectories.

### 2.4. Genetic Analysis

Genotyping of *TNF-α-308G/A* (rs1800629), *TNF-α-238G/A* (rs361525), *IL-6-174G/C* (rs1800795), *IL-6R 1073A/C Asp358Ala* (rs2228145), *IL-1B-31G/A* (rs1143627), *IL1A-2231* (rs3783521), *IL-10-1082C/T* (rs1800896), and *IL10-592 C/A* (rs1800872) SNPs was performed using allelic discrimination real-time PCR with pre-designed TaqMan assays (C__7514879_10 for rs1800629, C_2215707_10 for rs361525, C_1839697_20 for rs1800795, C_16170664_10 for rs2228145, C___1839944_10 for rs1143627, C___1839900_10 for rs3783521, C_174360_10 for rs1800896, and C-174363-10 for rs1800872) (Thermo Fisher Scientific, Waltham, MA, USA). The PCR protocol included a hot start at 95 °C for 10 min, followed by 40 cycles at 94 °C for 15 s and 60 °C for 1 min. Fluorescence detection was performed at 60 °C. Reactions were conducted in 10 μL volumes using the TaqMan Genotyping Master Mix on 96-well plates with a CFX96 instrument (Bio-Rad, Hercules, CA, USA). Control samples representing all possible genotypes, as well as a negative control, were included in each run.

### 2.5. Statistical Analysis

Chi-square analysis was applied to verify that the genotype distribution was in the Hardy–Weinberg equilibrium (HWE). Clinical scores were reported at T0 (admission) and T1 (after rehabilitation treatment) as means and standard deviations and the difference between the two time points was analyzed using repeated measures analysis with confidence intervals calculated for the mean differences. The presence of PTCS at T0 according to the GOAT was assessed by the percentage and corresponding 95% confidence interval (CI), and the presence of PTCS at T1 was assessed by the percentage of subjects who were confused at T0 and became not confused at T1 out of those who were confused at T0. The corresponding 95% CI was also calculated.

Clinical scores were also described at T0 and T1 within groups defined by genotypes, using median and interquartile ranges (25–75 percentiles), and the comparison of the distribution between genotypes was performed by non-parametric Kruskal–Wallis and Mann–Whitney tests.

The level of statistical significance was adjusted for multiple comparisons using Bonferroni correction by dividing the standard 5% threshold by the number of outcomes: 6 outcomes in Table 2, yielding a significance threshold of 0.05/6 = 0.008, and 14 outcomes in Supplementary Table S1, resulting in a threshold of 0.05/14 = 0.004.

The impact of cytokine genotypes on the delta scores of clinical scales (BI, POMA, FAB, ABS, DRS, derived at the end of the recovery treatment: T1–T0 values) was assessed by one-way analysis of variance (ANOVA). Furthermore, regression analyses were performed to correlate clinical delta values and genotypes adjusting for age, sex, presence of fever lasting more than one day requiring antibiotics (yes/no), the use of nonsteroidal anti-inflammatory (NSAIDs) drugs and corticosteroids (yes/no), and each baseline clinical score at T0.

Using these regression models, marginal means of delta clinical scores for groups defined by cytokine genotypes were obtained by setting values of independent variables equal to the mean of the whole data.

Allelic co-segregation analysis and association with rehabilitation outcomes were performed by SHEsisPlus online software (http://analysis.bio-x.cn, (accessed on 28 February 2025) [26,27]

## 3. Results

### 3.1. Cytokine Genetic Characterization

Single nucleotide polymorphisms (SNPs) in the promoter regions of genes encoding the pro-inflammatory TNF α, IL-6, and IL-1, as well as the anti-inflammatory cytokine IL-10, were investigated in all patients included in this study. Additionally, a non-synonymous SNP resulting in an Aspartate-to-Valine substitution at the cleavage site of the IL-6 receptor was analyzed. Genotype distributions are reported in Table 1 and show no deviations from the Hardy–Weinberg Equilibrium (HWE).

### 3.2. Outcome of the Rehabilitation Treatment

Clinical scores at T0 and T1 are presented in Table 2. A significant improvement (*p* < 0.001) was observed across all domains—functional, cognitive, and motor—following rehabilitation. Statistical significance was maintained after adjustment for multiple comparisons using the Bonferroni correction, with the corrected threshold set at *p* = 0.008 (0.05 divided by the number of outcomes). These encompassed enhancements in cognitive executive functions (FAB), reduction in agitation levels (ABS), improvements in motor skills (POMA), daily living activities (BI), and functional recovery (DRS). The emergence from PTCS (according to GOAT), defined as the proportion of subjects who were confused at T0 and became non-confused at T1, showed a lower bound of the 95% CI equal to 41.3%.

Clinical scores at T0 and T1, stratified by genotype groups, are reported in Supplementary Table S1. At T1, statistically significant differences were observed between subjects carrying the *TNFα-308* (GG) and (GA) genotypes in the BI, POMA, and PTCS scores (*p* = 0.005, *p* = 0.03, and *p* = 0.03, respectively). However, these differences did not remain statistically significant after applying the Bonferroni correction for multiple comparisons (adjusted *p*-value threshold = 0.004). Specifically, patients with the *TNFα-308* (GA) genotype exhibited poorer recovery, as indicated by lower BI and POMA scores, compared to those carrying the (GG) genotype. Similarly, a higher proportion of subjects with the *TNFα-308* (GA) genotype (75%) remained in a PTCS condition at T1, compared to only 17% of those with the (GG) genotype. The *IL-6-174*C/G polymorphism correlated significantly with BI and DRS scores at T0 (*p* = 0.02) and with PTCS frequency at T0 (*p* = 0.007). However, these associations also did not remain significant after applying the Bonferroni correction. In particular, the *IL-6-174* (GG) genotype was associated with lower BI scores compared to the (CG) and (CC) genotypes (*p* = 0.02). Similarly, higher DRS scores were observed in individuals with the (GG) genotype compared to those with (CG) and (CC) (*p* = 0.02). The percentage of patients with PTCS was also higher among (GG) carriers compared to those with (CG) (*p* = 0.2) and (CC) (*p* = 0.02) genotypes. However, no statistically significant correlation between *IL-6-174* genotypes and PTCS frequency at T1 was observed. 

The *IL-6R 1073*A/C polymorphism was significantly associated with FAB scores at both T0 (*p* = 0.003)—which remained significant after the Bonferroni correction—and T1 (*p* = 0.03), as well as with DRS at T1 (*p* = 0.002), which also remained significant after correction. Specifically, individuals with the *IL-6R* (AA) genotype had lower FAB scores at T0 compared to those with (AC) (*p* = 0.04) or (CC) (*p* = 0.003) genotypes, and this difference persisted after rehabilitation.

Lower GOSE scores were also observed in *IL-6R* (AA) carriers at both T0 and T1 compared to those with the (AC) and (CC) genotypes; the difference reached statistical significance only at T1, specifically when comparing (AA) to (AC) genotype (*p* = 0.003).

**Figure 1:**
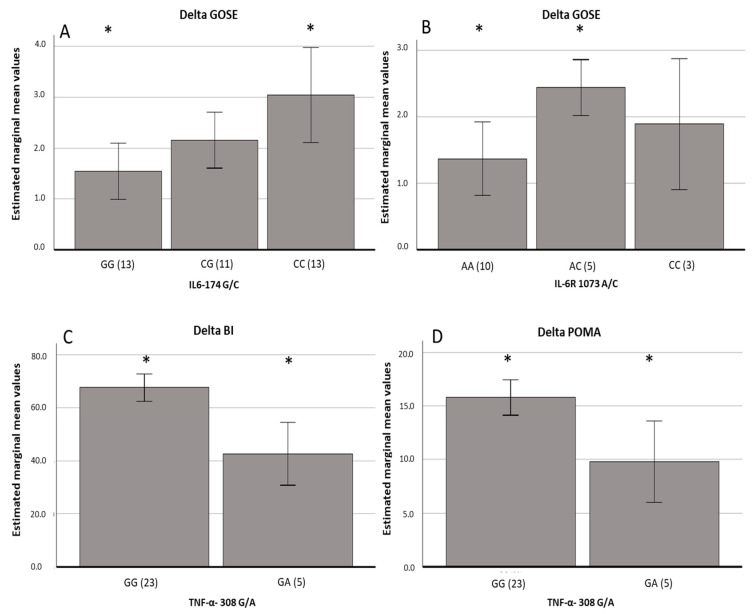
Genotype distributions of *IL-6-174*(G/C), *IL-6R 1073*(A/C), and *TNFα-308*(G/A). Legend: genotype distribution of *IL-6-174*(G/C), *IL-6R 1073*(A/C), and *TNFα-308*(G/A) impact on rehabilitation outcome measured by mean delta values (T1–T0) of GOSE, BI, and POMA scores. (**A**): Mean delta GOSE values within groups defined by *IL-6-174* G/C genotype. Marginal means obtained by linear regression analysis setting GOSE at admission = 3.68, GG vs. CC, * *p* = 0.02. (**B**): Mean delta GOSE values within groups defined by *IL-6R 1073*A/C genotype. Marginal mean obtained by linear regression analysis setting GOSE at admission = 3.68, AA vs. AC, * *p* = 0.01. (**C**): Mean delta BI values within groups defined by *TNFα-308* G/A genotype. Marginal mean obtained by linear regression analysis setting BI at admission = 22.93, GA vs. GG, * *p* = 0.001. (**D**): Mean delta POMA values within groups defined by *TNFα-308* G/A genotype. Marginal mean obtained by linear regression analysis setting POMA at admission= 10.36 GA vs GG, * *p* = 0.01. Furthermore, the following covariates were added in the linear regression models and were evaluated at the following values: Age = 45.75, Sex M = 81.75, number of infectious events: 28.7%, use of NSAIDs, and corticosteroids: 17.8%. Error bars: ±2 SE.

Genotype distributions of all SNPs in cytokine genes were analyzed in terms of their relationship with rehabilitation outcomes across different functional domains. Subjects carrying the *IL-6-174*(GG) genotype showed lower predicted delta GOSE scores than those with the (CC) genotype (*p* = 0.02) (Figure 1A, Table 3). Similarly, individuals with the *IL-6R 1073* (AA) genotype had a lower predicted delta GOSE score than those carrying the (AC) genotype (*p* = 0.01) (Figure 1B, Table 3). No associations were observed between the predicted delta GOSE value and age, sex, systemic infections with fever, or treatments with NSAIDs or corticosteroids. Furthermore, no significant relationship was found between these SNPs and delta values in other clinical scales.

The *TNFα-308* genotype showed a significant impact on predicted mean delta values of both the Barthel and POMA indexes. Specifically, patients carrying the *TNFα-308* (GA) genotype exhibited less improvement than those carrying the (GG) genotype both for BI (*p* = 0.001) (Figure 1C, Table 3) and POMA scores (*p* = 0.01) (Figure 1D, Table 3). In these cases, improvements in both the BI and POMA scores were associated with younger age and lower BI and POMA scores at admission but were not influenced by systemic events or treatment with NSAIDs or corticosteroids.

Finally, no significant correlations were found between any of the other clinical parameters analyzed and the remaining cytokine gene SNPs.

Notably, when evaluating the presence of PTCS at T1, the persistence of a confusional state was still observed in 3 out of 4 individuals (75%) carrying the *TNFα-308* (GA) genotype but only in 2 out of 12 (17%) individuals with the *TNFα-308* (GG) genotype. The chi-square test on the absence of association between the genotype and confusion state after rehabilitation was evaluated on the subset of patients who were confused at admission, showing a statistically higher risk of maintaining confusion at T1 among the carriers of the *TNFα-308* (GA) genotype (*p* = 0.03)

### 3.3. Co-Segregation Analysis

Since the IL-6–IL6R interaction is fundamental to the IL-6 signaling, a co-segregation analysis was performed to evaluate how *IL-6-174* G/C and *IL-6R 1073* A/C alleles may impact the rehabilitation outcome of TBI patients. Significant lower GOSE delta scores were seen in subjects carrying both the *IL-6-174* (G) and the *IL-6R 1073* (A) alleles (beta value = −0.656, *p* = 0.004); conversely patients with the complementary *IL-6-174* (C) and *IL6R 1073 (C)* alleles showed a higher GOSE delta score (beta = 1.105, *p* = 0.002) (Table 4).

## 4. Discussion

The aim of this pilot study was to assess whether genetic polymorphisms in pro and anti-inflammatory cytokines contribute to inter-individual variability could account for differences in individual rehabilitation outcomes among of patients with a subacute TBI.

Rehabilitation outcomes were assessed using both the GOSE, commonly employed in TBI research, and a set of domain-specific scales evaluating cognitive, motor, and functional recovery (e.g., FAB, ABS, POMA, BI, DRS). While most prognostic studies rely solely on GOSE due to its simplicity, our study leveraged the rehabilitation setting to implement a more granular and multidimensional outcome assessment. This approach not only confirmed significant functional improvements following rehabilitation but also revealed interindividual variability in recovery trajectories, suggesting a role for genetic factors. Among the genetic variants analyzed, the *IL-6-174* (GG) and *IL-6R 1073* (AA) genotypes were associated with poorer recovery. Individuals with these genotypes exhibited greater cognitive and functional impairment at the baseline and lower global improvement after rehabilitation, as reflected by the GOSE score. These findings support a possible role of IL-6 pathway polymorphisms in modulating the neuroinflammatory response and influencing rehabilitation responsiveness.

Although the *TNFα-308* G/A polymorphism was not associated with the overall GOSE outcome, the (GA) genotype was linked to reduced recovery in specific functional domains, particularly autonomy and motor performance (BI and POMA). Moreover, PTCS was more frequent after rehabilitation in (GA) carriers, indicating a potential role of TNF-α in neurobehavioral complications post-TBI.

These findings are consistent with previous studies associating the *TNFα-308* (GA) genotype with more severe brain injury [28] and poorer treatment and rehabilitation outcomes in TBI patients. These results also reinforce both experimental and clinical evidence indicating that neuroinflammation impacts clinical outcomes following TBI, with TNF-*α* playing an important role in this process [9,29].

The association between the *TNFα-308* G/A polymorphism and TBI outcome is biologically plausible, as this genetic variation influences TNF-*α* production in response to stimuli such as TBI. The *TNFα-308* G/A polymorphism is located in the promoter region of the gene, where it modulates transcription, with the *TNF-α-308*(A) allele being associated with significantly higher TNF-α production [30]. An increase in TNF-α production results in systemic and local inflammation [31,32,33], thus influencing neuroinflammation as well [9,34,35,36].

Increased TNF-α and IL-6 plasma concentrations have been associated with reduced physical performance and a diminished ability to perform everyday tasks [37,38] and are predictors of disability in the elderly population [39]. Furthermore, a significant interaction of *TNF-α-308* SNP and the effect of exercise on physical performance was reported in elderly women [40].

Contradictory results exist in the scientific literature regarding the *IL-6-174* (GG) genotype and its association with TBI [3]. While some authors have found no correlation between these variables [9], others have reported a positive influence of this genotype on TBI outcomes [41], and yet, others have observed worse outcomes in individuals carrying the same genotype [42]. These discrepancies might be explained by the dual role of IL-6, which can act as a pro-inflammatory cytokine that increases cerebral edema but may also promote neurogenesis and drive microglia toward an anti-inflammatory phenotype [43]. Some studies associate elevated IL-6 levels with poorer outcomes, whereas others report either a beneficial effect or no significant correlation at all [44].

Our findings support the hypothesis of a negative role of the *IL-6-174* (GG) genotype on the overall outcome of TBI.

To further clarify the role of IL-6 in TBI, we expanded our evaluation to encompass the combined effects of SNPs in the *IL-6* and *IL-6R* genes. Notably the *IL-6R 1073* (A) variant is associated with more efficient cleavage of the membrane-bound IL-6R, resulting in a reduced number of functional membrane-bound IL-6R, accompanied by higher levels of circulating soluble IL-6R (sIL-6R) and correlates with an enhanced response to IL-6 cytokine [45]. Indeed, our results show a correlation between both the *IL-6-174* (GG) and the *IL-6R 1073* (AA) genotypes with worse GOSE values, thus showing a combined effect of *IL-6-174-IL6R 1073* (G-A) alleles on poorer GOSE outcomes. Taken together, these results suggest that the *IL6-174* (G) and *IL6R 1073* (A) polymorphisms may influence recovery trajectories in TBI patients by enhancing the inflammatory response. The combination of increased IL-6 production, linked to the G-variant of *IL6-174* [40,46], and increased sensitivity to this cytokine, linked to the A-variant of *IL-6R 1073* [45], could hinder recovery as a consequence of sustained inflammation. 

In this context, it is noteworthy to underline that the immunological effects resulting from IL-6 binding to its receptors are extremely complex. Thus, IL-6 is the major pro-inflammatory member of the IL-6 family, and it signals via glycoprotein 130 (gp130) and either cell membrane-bound or soluble receptors. IL-6 binding to the cell-bound IL-6R (also known as classic signaling) has homeostatic functions. IL-6 binding to soluble IL-6R(sIL-6R) (also known as trans-signaling) promotes a pro-inflammatory response, which is inactivated by the formation of a ternary IL-6/sIL-6R/sgp130 complex. The predominance of one pathway over the other may vary among individuals and clinical contexts, potentially accounting for the heterogeneity of results observed across studies. Indeed, the *IL-6R 1073* (A) variant shows a more efficient cleavage of the membrane-bound IL-6R, resulting in a reduced number of functional membrane-bound IL-6R, accompanied by higher levels of circulating sIL-6R [45]. Furthermore, the ratio between binary IL-6/sIL-6R and the ternary IL-6/sIL-6R/sgp130 complexes indicates the amount of active, proinflammatory IL-6. This balance between IL-6, sIL-6R, and sgp130 allows the evaluation of either the trans-signaling or inhibition of IL-6 activity, depending on whether the binary or ternary complex predominates. While our findings support the hypothesis that the *IL-6-174* (G) allele, in combination with increased cytokine sensitivity due to the (A) variant of the *IL-6R 1073*, has a negative impact on the overall outcome of TBI, these parameters will need to undergo in-depth analyses to better understand the role played by the IL-6/IL-6R in TBI [47]. Individual responses may vary, and further studies are needed to fully understand these effects in the context of brain injury. 

Complex interactions occur between different cytokines, thus, different combinations of alleles across cytokine genes result in different effects. In this context, we suggest that the *TNF-α-308*, *IL-6-174*, and *IL-6R 1073* SNPs combination contributes to an increased risk of poorer rehabilitation outcomes in TBI patients as a result of their ability to amplify neuroinflammation. Prolonged or excessive inflammatory responses following TBI can reduce neuroplasticity, delay tissue repair, and exacerbate secondary injury mechanisms, ultimately affecting functional recovery [48]. Interestingly, our findings suggest a differential impact of cytokines SNPs on specific rehabilitation outcomes: the *TNF-α-308* (GA) genotype appears to be more strongly associated with impairments in motor and functional independence domains, as reflected by lower BI, POMA scores, and the persistence of PTCS. This likely reflects the TNF-*α* role in disrupting neuromuscular function and motor coordination through inflammation-mediated damage [49,50]. Conversely, IL-6/IL-6R and IL-6/sIL-6R trans-signaling appear to influence broader aspects of global prognosis, as indicated by GOSE scores. Given IL-6 dual role in neuroinflammation and neuroprotection [51], its dysregulation might lead to a more widespread impact on brain homeostasis, affecting multiple recovery processes beyond specific functional domains. 

Overall, cytokine gene polymorphisms may contribute to inter-individual variability in post-TBI recovery. In particular, the association of *TNFα-308*(GA) genotype with reduced functional improvement may be due to a more sustained pro-inflammatory response in line with evidences linking *TNFα-308*(GA) variant to increased cytokine expression and worse clinical outcomes in TBI patients [52]. Notably, TNFα has a context-dependent role in neuroinflammation: activation of TNF receptor 1 (TNFR1) promotes inflammation and cell death, while TNFR2 may mediate neuroprotective effects [53]. This dual function may help explain why non-selective anti-TNF strategies have failed in clinical trials. In this light, identifying patients with pro-inflammatory genetic profiles—such as *TNFα-308*(GA) carriers—could guide more targeted therapeutic approaches aimed at selectively modulating TNFα signaling.

Recent frameworks for post-TBI immunomodulation emphasize the importance of accounting for individual biological variability, including genetic susceptibility, injury timing, and mechanism [52]. Our results support this view, suggesting that cytokine-related polymorphisms could serve not only as prognostic markers but also to stratify patients for future trials testing anti-cytokine or neuroprotective agents.

Although our study was not designed to evaluate therapeutic efficacy, the association between genotype and outcome highlights the potential of integrating genetic stratification with anti-inflammatory therapies in the post-acute phase of TBI. This approach may be particularly valuable in rehabilitation settings, where persistent inflammation remains a modifiable factor and could be addressed through individualized treatment strategies.

Limitations: This study has some limitations that must be acknowledged. First, the small sample size limits the statistical power of the analysis and classifies this work as a pilot study. While the findings are preliminary, they offer a valuable starting point for future investigations in larger and more diverse cohorts. Another limitation is the absence of cytokine level measurements in cerebrospinal fluid (CSF), which would have provided a deeper mechanistic insight into the observed genotype–phenotype associations. However, due to ethical and logistical constraints—particularly related to the rehabilitative setting in which this study was conducted, where CSF sampling is not routinely feasible in the absence of ventricular drains—this was not possible in our cohort. Future studies should aim to integrate molecular data—including cytokine expression profiles from CSF —with clinical and genetic information to clarify the biological underpinnings of post-TBI recovery. Despite these limitations, our results are consistent with the previous literature showing that the investigated polymorphisms influence cytokine expression levels and inflammatory responses. This supports the biological plausibility of our findings and reinforces the need for further translational research in this area.

## 5. Conclusions

This study provides promising insights into the role of individual genetic profiles in post-TBI prognostic models. Incorporating genetic information on inflammatory pathway polymorphisms may improve patient stratification by recovery potential and enhance the accuracy of clinical outcome predictions. Patients with variants linked to heightened inflammatory responses could benefit from targeted anti-inflammatory treatments alongside standard rehabilitation. Early identification of these genetic risk factors may also optimize resource allocation by tailoring care intensity and duration. Importantly, these findings encourage further genetic research to better understand the molecular mechanisms behind recovery variability, advancing personalized medicine approaches in TBI care and paving the way for more precise and effective interventions.

## Figures and Tables

**Table 1:** Cytokine genotype distributions in 28 TBI patients.

	Genotype	n (%)	HWE *p*-Value
*IL1A-2231 G/A*	G G	14 (50.0)	0.79
	A G	12 (42.9)	
	A A	2 (7.1)	
* IL-1B-3 1G/A *	G G	7 (25.0)	0.23
	G A	17 (60.7)	
	A A	4 (14.3)	
* IL-6-174 G/C *	G G	13 (46.4)	0.51
	G C	11 (39.3)	
	C C	4 (14.3)	
*IL-6R 1073 A/C*	A A	10 (35.7)	0.45
	A C	15 (53.6)	
	C C	3 (10.7)	
* IL-10-1082 T/C *	T T	10 (35.7)	0.28
	T C	11 (39.3)	
	C C	7 (25.0)	
* IL10-592 G/T *	G G	21 (75.0)	0.45
	G T	7 (25.0)	
* TNF-α-238 G/A *	G G	26 (92.9)	0.84
	G A	2 (7.1)	
* TNF-α-308 G/A *	G G	23 (82.1)	0.60
	G A	5 (17.9)	

*p*-value is related to Hardy–Weinberg equilibrium analysis. n = absolute number of subjects.

**Table 2:** Pre-(T0) and post-treatment (T1) evaluation of outcome scales.

	Score	Delta Values		
	Mean (sd)	Mean	95%CI	*p*-Value
GOSE T0	3.7 (0.5)	2.0	1.65:2.35	<0.001
GOSE T1	5.7 (1.2)			
FAB T0	10.6 (5.5)	3.3	2.01:4.58	<0.001
FAB T1	13.9 (5.4)			
ABS T0	22.9 (6.9)	−5.8	−7.8:−3.8	<0.001
ABS T1	17.2 (4.1)			
DRS T0	14.8 (4.5)	−9.7	−11.5:−7.9	<0.001
DRS T1	5.10 (3.9)			
BI T0	22.9 (25.2)	63.2	52.9:73.5	<0.001
BI T1	86.1 (19.9)			
POMA T0	10.4 (9.9)	14.7	11.1:18.4	<0.001
POMA T1	25.1 (6.0)			
	(n of subjects) yes/no	success rate	95% CI	
Presence of PTCS at T0 according to GOAT	16/12	11/16	41.3%:88.9%	
Presence of PTCS at T1 according to GOAT	5/11			

The following are reported as means and standard deviations at T0 and T1: GOSE: Glasgow Outcome Scale Extended; FAB: Frontal Assessment Battery; ABS: Agitated Behavior Scale; DRS: Disability Rating Scale; BI: Barthel Index; and POMA: Performance-Oriented Mobility Assessment. Delta means and their corresponding 95% confidence interval (CI) were also calculated. *p*-values were obtained through paired data analysis. The presence of a post-traumatic confusional state (PTCS) was assessed using the Galveston Orientation and Amnesia Test (GOAT). At T0, the presence of PTCS as determined by the GOAT is reported as the ratio of PTCS yes/no cases, as well as the corresponding percentage. At T1, the PTCS presence is reported as the number of subjects who remained confused out of those who were confused at T0. The success rate is defined as the proportion of subjects who became not confused at T1 among those who were confused at T0, along with the corresponding 95% CI.

**Table 3:** Linear regression analyses.

	Delta GOSE			
	Beta Value	95%CI		*p*-Value
(Intercept)	1.56	−2.52	5.64	0.43
Age	−0.01	−0.03	0.01	0.27
GOSE at admission	0.58	0.39	1.47	0.16
Sex [M vs. F]	−0.01	−0.99	0.96	0.97
Systemic infections [yes vs no]	0.11	−0.75	0.97	0.79
NSAIDs and corticosteroids [yes vs no]	−0.58	−1.64	0.48	0.27
* IL-6-174 * [GC vs. CC]	−0.89	−1.95	0.17	0.09
* IL-6-174 * [GG vs. CC]	−1.49	−2.74	−0.25	0.02
(Intercept)	−0.36	1.55	−0.23	0.82
Age	0.01	0.01	0.51	0.62
GOSE at admission	0.42	0.40	1.04	0.31
Sex [M vs. F]	−0.11	0.45	−0.25	0.80
Systemic infections [yes vs. no]	0.07	0.39	0.18	0.86
NSAIDs and corticosteroids [yes vs. no]	0.32	0.41	0.77	0.45
* IL-6R 1073 * [AC vs. AA]	1.07	0.37	2.91	0.01
* IL-6R 1073 * [CC vs. AA]	0.52	0.59	0.87	0.39
(Intercept)	91.02	68.99	113.05	<0.001
Age	−0.59	−0.91	−0.28	<0.001
BI at admission	−0.70	−0.91	−0.50	<0.001
Sex [M vs. F]	−4.55	−18.86	9.76	0.52
Systemic infections [yes vs. no]	−1.70	−14.36	10.95	0.78
NSAIDs and corticosteroids [yes vs. no]	−2.72	−16.31	10.85	0.68
* TNF-alpha 308 * [GA vs. GG]	−24.95	−38.81	−11.09	0.001
(Intercept)	23.01	15.95	30.07	<0.001
Age	−0.13	−0.24	−0.05	0.02
POMA admission	−0.77	−0.96	−0.57	<0.001
Sex [M vs. F]	1.53	−3.19	6.24	0.51
Systemic infections [yes vs. no]	−3.46	−7.67	0.74	0.10
NSAIDs and corticosteroids [yes vs. no]	2.27	−2.03	6.58	0.28
* TNF-alpha 308 * [GA vs. GG]	−5.99	−10.45	−1.55	0.01

The Beta value estimate represents the impact on the average of the outcomes (delta GOSE, delta BI, Delta POMA) of a 1 increment in each independent continuous variable (Age GOSE, BI, POMA at admission); or the presence of the binary variable (Sex, Systemic infections, NSAIDs and cortico-steroids, *IL-6-174*; *IL-6R 1073*, *TNF-α 308*) with respect to the reference.

**Table 4:** Frequencies of the *IL-6-174G/C* and *IL-6R 1073* A/C SNP (freq. = n/56) showing co-segregation distribution in 28 subacute TBI patients.

Allelic Co-Segregation Association with Delta GOSE			
*** IL-6-174 *** ** G/C **	* IL-6R 1073 * A/C	Beta Value	*p*-Value
C	A	0.22	0.47
G	A	−0.66	0.004
G	C	0.09	0.74
C	C	1.11	0.002

The Beta value represents the impact on the average of the delta GOSE.

## Data Availability

The data presented in this study are available upon request from the corresponding author.

## Supplementary Materials

The following supporting information can be downloaded at https://www.mdpi.com/article/10.3390/cells14141056/s1. Table S1: Clinical scores at T0 and T1, within groups defined by Cytokine Genotype. Description of data in Supplementary Table S1: The distribution of the Cytokine Genotype in 28 TBI patients was reported as an absolute number (n) and percentages (%). Pre (T0) and post (T1) treatment evaluation of the outcomes scales of BI: Barthel Index; POMA: Performance Oriented Mobility Assessment, FAB: Frontal Assessment battery; ABS: Agitated Behaviour Scale; DRS: Disability Rating Scale; GOSE: Glasgow outcome scale are reported as median and 95% Confidence of interval (CI). The presence of a post-traumatic confusional state (PTCS) was assessed using the Galveston Orientation and Amnesia Test (GOAT). The presence of PTCS at T0 according to the GOAT is reported as the number of subjects with PTCS (n1) and by percentage of n1/n (% of n). The presence of PTCS at T1 is reported as the number of subjects who were confused at T1 (n2) out of those who were confused at T0 (n1) (% of n1). * = statistically significant *p*-value before correction for multiple comparisons (uncorrected) * = statistically significant *p*-value also after Bonferroni correction for multiple comparisons.

## Abbreviations

TBI: traumatic brain injury; TNF: tumor necrosis factor; IL: interleukin; GOSE: Glasgow Outcome Scale Extended; POMA: Performance-Oriented Mobility Assessment; SNP: single nucleotide polymorphism; IRU: intensive rehabilitation unit; FAB: Frontal Assessment Battery; ABS: Agitated Behavior Scale; BI: Barthel Index; DRS: Disability Rating Scale; PTCS: post-traumatic confusional state; LCF: level of cognitive function; GOAT: Galveston Orientation and Amnesia Test; PCR: polymerase chain reaction; HWE: Hardy–Weinberg equilibrium; NSAIDs: nonsteroidal anti-inflammatory drugs.
